# PReS13-SPK-1107: Development of an academic pediatric rheumatology program

**DOI:** 10.1186/1546-0096-11-S2-I33

**Published:** 2013-12-05

**Authors:** R Laxer

**Affiliations:** 1 Pediatrics and Medicine, The Hospital for Sick Children and the University of Toronto, Toronto, Canada

## 

The development of the Pediatric Rheumatology Program in Toronto began in 1984 with the recruitment of 3 junior but well-trained pediatric rheumatologists. Their goal, to create the finest pediatric rheumatology program in the world, was predicated on the need to both create and disseminate new knowledge. This required that all the elements of a strong academic program would be supported; (1) scholarly clinical care; (2) education at all levels including medical student, resident, fellow and continuing education; and (3) research, both clinical and basic. Early salary support from The Arthritis Society was critical to the recruitment and the hiring of a clinical fellow.

Recognition of the expertise of the individual staff provided opportunities to pursue specific interests in a collegial and non-competitive environment. The shared vision was embraced such that individual goals and successes all contributed to the greater good and allowed each member to share in the successes of the others; each achievement not only advanced an individual's program but the entire division benefitted. Early provision of resources by the institution (space, nursing, rehabilitation, social work) provided the opportunity to rapidly build the clinical program. The development of sub-specialty interests was encouraged leading to a variety of specific clinics that built clinical experience and expertise, provided large cohorts for clinical research and allowed trainees to see a large number of patients with rare diseases even if they had otherwise limited exposure to rheumatology. The strong Research Institute affiliated with the hospital provided excellent scientific support for both clinical and basic science research endeavours. Presentation and publication of our work and international meetings gave us early credibility and highlighted training opportunities. To date, over 70 trainees from 27 countries have come through the program, and graduates continue to promote the program as a centre of excellence for clinical and research training.

The presentation will review the development of the program and highlight the elements that have allowed the Toronto program to remain one of the leading academic programs for pediatric rheumatology in the world.

## Disclosure of interest

R. Laxer Grant/Research Support from: Novartis.

